# The Reaction of Dimerization by Itself Reduces the Noise Intensity of the Protein Monomer

**DOI:** 10.1038/s41598-019-39611-6

**Published:** 2019-03-04

**Authors:** Feng-You Liu, Shih-Chiang Lo, Che-Chi Shu

**Affiliations:** Department of Chemical Engineering and Biotechnology, National Taipei University of Technology, Taipei City, Taiwan R.O.C.

**Keywords:** Biochemical reaction networks, Gene regulatory networks

## Abstract

Because of the small particle number of intracellular species participating in genetic circuits, stochastic fluctuations are inevitable. This intracellular noise is detrimental to precise regulation. To maintain the proper function of a cell, some natural motifs attenuate the noise at the protein level. In many biological systems, the protein monomer is used as a regulator, but the protein dimer also exists. In the present study, we demonstrated that the dimerization reaction reduces the noise intensity of the protein monomer. Compared with two common noise-buffering motifs, the incoherent feedforward loop (FFL) and negative feedback control, the coefficient of variation (COV) in the case of dimerization was 25% less. Furthermore, we examined a system with direct interaction between proteins and other ligands. Both the incoherent FFL and negative feedback control failed to buffer the noise, but the dimerization was effective. Remarkably, the formation of only one protein dimer was sufficient to cause a 7.5% reduction in the COV.

## Introduction

Gene expression in cells is subject to stochastic fluctuations. The population heterogeneity caused by intracellular stochasticity plays a critical role in the decisions related to the cellular fate, such as the onset of conjugation^1,2^, the switch of intracellular states^3,4^, the detection of signal^5,6^, the synchronization of gene expression^7,8^, and so on^9–11^. Note that cell-to-cell variations cannot be avoided by simply applying genetically identical cells in a homogeneous environment. The origin of intracellular stochasticity is in cellular processes, such as transcription and translation. Given the high level of intracellular stochasticity in gene regulation, it is not difficult to imagine that many endogenous noise-buffering motifs maintain stability. Among them, the best-known motifs are negative feedback control^12^ and the incoherent feedforward loop (FFL)^13^. These two motifs have been reported to attenuate noise^12–15^ and examined in the present study. It is our aim to discover a novel motif, the reaction of dimerization, and to evaluate its noise-buffering ability.

One common type of negative feedback control comprises a downstream protein repressing its own gene by inhibiting transcription^16,17^. Negative feedback control reacts directly with upstream elements to ensure the attenuation of noise, regardless of the complexity of the reaction network. However, it does not effectively attenuate noise of the system with a short response time. Besides, a transcriptional or translational delay may escalate this drawback. As for incoherent FFL, its traces have been found in various organisms, including both eukaryotes and prokaryotes. The incoherent FFL usually includes a transcription factor (TF), which activates the expression of miRNA (or sRNA) and the target RNA^18^. “Incoherent” describes the different influences of the TF and miRNA (or sRNA) on the target RNA. In eukaryotes, the TF activates the expression of the target RNA^19^, but miRNA represses the translation of the target protein^13^.

In this study, we didn’t include the coherent FFL because only a certain type of it can reduce the noise^20^. For incoherent FFL, we examined the system in prokaryotes^21^. In prokaryotes, sRNA instead of miRNA is the main posttranscriptional regulator. It binds to the target RNA to repress gene expression^2,21^. The sRNA fluctuates in the same direction as that of the target RNA because the same TF activates both of them. As sRNA represses the expression of the target protein, it buffers the protein noise inherited from the TF^13^. Consequently, the incoherent FFL attenuates little noise from other sources. We attempted to demonstrate that the dimerization reaction is free from the aforementioned problems encountered by feedback control or the incoherent FFL.

## Results

**Figure 1:**
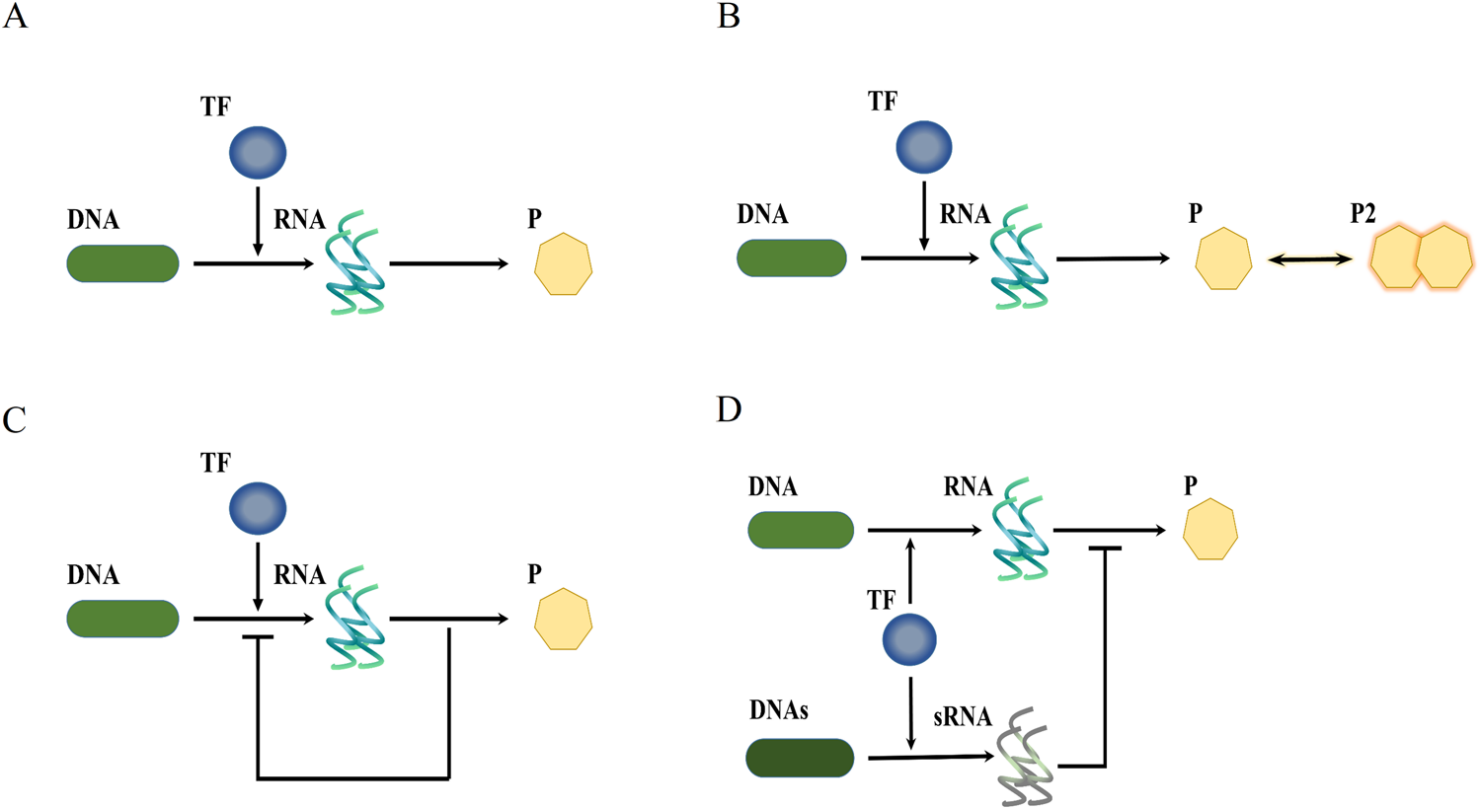
The four reaction networks. (**A**) Basic gene expression without a noise-buffering motif, (**B**) gene expression with a dimerization reaction, (**C**) gene expression with feedback control, and (**D**) gene expression with the incoherent FFL.

Some works in literature^16,22–24^ involved dimer as a regulator in a system with the feedback control. Their approaches are fundamentally different from ours. They discussed the influence of multiple regulatory binding sites on the noise attenuation in the feedback control. We aim to illustrate that the dimerization alone is sufficient to attenuate the noise of protein monomer. The feedback control is not needed in our work. We investigated the four cases shown in Fig. 1. Figure 1A demonstrates typical gene expression without any noise-buffering motif. In Fig. 1B, the target protein is allowed to undergo dimerization. The monomer of the target protein is the regulatory determinant and we investigated the ability of dimerization to attenuate the noise of the protein monomer. We also examined two other noise-buffering motifs. One is negative feedback control, shown in Fig. 1C; the other is the incoherent FFL, illustrated in Fig. 1D.

### Dimerization attenuated the noise of the protein monomer

**Figure 2:**
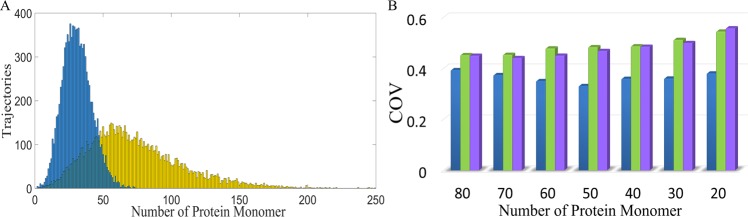
Dimerization attenuated the noise of the protein monomer. (**A**) Distribution of the protein monomer. Yellow bars represent the case without a noise-buffering motif. After dimerization, the distribution of the protein monomer became much sharper (blue bars). (**B**) COV of the protein monomer. The blue bars represent the case with the dimerization reaction, green bars the case with feedback control, and purple bars the case with the incoherent FFL.

In many natural systems, protein monomer is the minimal functional unit but the dimer also coexists^25–29^. Dimerization may play a crucial role in attenuating the noise of the monomer. To examine this function of dimerization, we conducted a stochastic simulation of reaction networks in Fig. 1A,B. Figure 2A demonstrates the stationary distribution of the protein monomer. The yellow bars represent the case in Fig. 1A, and the blue bars the case in Fig. 1B. The coefficient of variation (COV) of the free target protein is 0.4725 for the case without dimerization (yellow) and 0.3309 for the case where half of the protein monomers became dimers (blue). Dimerization notably reduced the noise of the protein monomer.

### Dimerization reduced more noise than the other two noise-buffering methods

Three noise-buffering motifs were studied: dimerization (Fig. 1B), negative feedback control (Fig. 1C), and the incoherent FFL (Fig. 1D). The COV of the protein monomer was shown in Fig. 2B; the blue bars represent the case with the dimerization reaction, the green bars the case with negative feedback control, and the purple bars the case with the incoherent FFL. Application of the noise-buffering motif reduced the level of the protein monomer and the *x*-axis represents the number of protein monomers. To achieve these values, we performed the following. For the case with the dimerization reaction, we adjusted the dissociation rate constant, *k*_*RPP*_ in Table S1. For the other two noise-buffering motifs, we tuned the parameters of the feedback and feedforward strength, which are *K* and *N* in Table S1, respectively. For the case without a noise-buffering motif, the COV was 0.4725 with averaged particle number of protein monomers as 100. Both negative feedback control (Fig. 1C) and the incoherent FFL buffered noise when the particle number of protein monomer is not too low, but the case with the dimerization reaction always exhibited the lowest COV.

### Dimerization greatly reduced random fluctuations when the protein level was maintained

**Figure 3:**
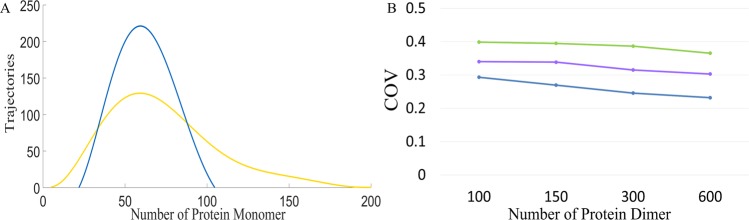
While applying noise-buffering methods, we also adjusted the transcription rate to maintain the same level of the protein monomer. (**A**) Distribution of the protein monomer. The yellow curve denotes the case without a noise-buffering motif, and the blue curve denotes the cases with the reaction of dimerization. (**B**) COV of the protein monomer. Blue curve denotes the case with the reaction of dimerization, green curve the case with feedback control, and purple curve the case with incoherent FFL.

Because noise-buffering methods reduced the protein level, they inevitably altered the noise intensity. To avoid this effect, we adjusted the transcription rate to maintain the level of the protein monomer at 100 particles per cell from the aspect of deterministic steady-state analysis; this adjustment was applied to obtain all the results shown in Fig. 3. With the stochastic simulations, the mean particle number of protein deviated from 100 because of the nonlinearity. This phenomenon has been well discussed in the literature^5,6,30^, especially the Grima’s work^30^, which discussed different kinds of dimerization reactions and their effects on the mean value.

Figure 3A shows the distribution of the protein monomer. The yellow curve represents the case of gene expression without a noise-buffering motif, and the blue curve represents that with the dimerization reaction. The COV of the protein monomer changed from 0.4725 to 0.2456 after dimerization and the particle number of dimers is 300. We subsequently examined how the number of dimers affects noise attenuation. To achieve the values shown on the *x*-axis in Fig. 3B, we performed the following. We tuned the transcription rate in order to have the number of proteins as 100 plus twice the particle number specified on the *x*-axis in Fig. 3B. We then accounted for dimerization and adjusted the dissociation rate constant, *k*_*RPP*_ in Table S1, to make the number of protein monomers to 100. For the other two noise-buffering motifs, we adjusted the feedback and feedforward strength, *K* and *N* in Table S1, to make the number of protein monomers to 100. Figure 3B demonstrates the COV of the protein monomer for various numbers of dimers. The blue curve denotes the case with dimerization, the green represents the case with negative feedback control, and the purple denotes the case with the incoherent FFL. Remarkably, the case with dimerization always exhibited the lowest COV.

### Dimerization buffered the noise of the system with direct interaction between proteins and other ligands

**Figure 4:**
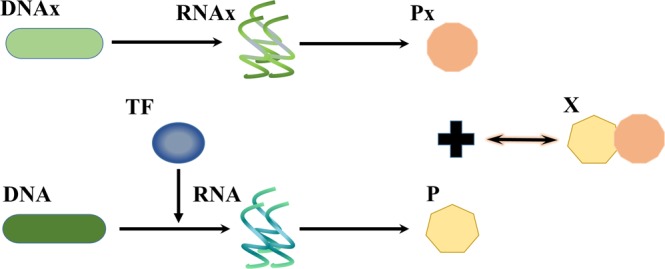
Reaction networks with direct interaction between the protein monomer and ligand. Based on the system in Fig. 1A, we further accounted for the direct interaction between the protein monomer and ligand Px.

**Figure 5:**
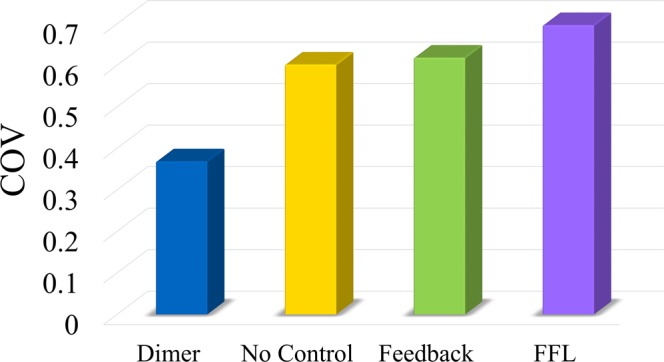
Dimerization buffered the noise in the system with direct interaction between the protein and the ligand. Blue bar represents the case with the dimerization reaction, yellow bar the case without a noise-buffering motif, green bar the case with feedback control, and purple bar the case with the incoherent FFL. The case with the dimerization reaction exhibited the lowest COV of the protein monomer.

It is important to understand how the noise-buffering motifs apply to a system with a direct interaction between proteins and other ligands. We then introduced a peptide ligand, Px, which reacts with the protein monomer to deactivate it. The reaction network that includes Px is illustrated in Fig. 4. It is the system of gene expression shown in Fig. 1A with the additional reaction between the protein and the ligand, Px. For systems with noise-buffering motifs shown in Fig. 1B–D, the corresponding reaction networks with Px were shown in Figs S1–S3. To better observe the noise from the protein-ligand interaction, we adjusted the transcription and translation rate constants of the TF to reduce the noise from gene expression. Specifically, we adjusted *k*_*Rt*_ and *k*_*T*_ in Table S1 to 6 × 10^−2^ and 4 × 10^−2^, respectively. With all other parameters at nominal values, we obtained the COV of protein monomers as shown in Fig. 5. The dimerization reaction effectively attenuated the noise though both the incoherent FFL and feedback control failed.

### The dimerization reaction attenuates noise from various sources

**Figure 6:**
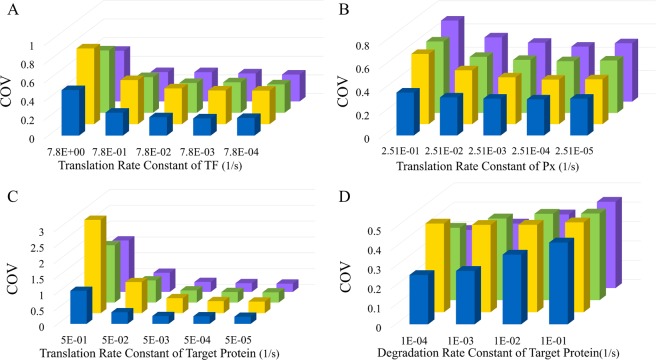
The dimerization reaction attenuates noise from various sources. The y-axis is the COV of the protein monomer. The blue bars indicate the case with the dimerization reaction, yellow bars the case without a noise-buffering motif, green bars the case with feedback control, and purple bars the case with the incoherent FFL. By adjusting the translation rate, we manipulated the noise of (**A**) TF, (**B**) Px, or (**C**) target protein. (**D**) We also examined the influence of the degradation rate of the target protein. For all four scenarios, we keep the level of TF, Px, or target protein the same by adjusting the transcription rate accordingly.

We then examined various noise intensity from different sources. We manipulated the noise intensity of TF or Px by altering its translation rate constant. To keep the same level of the TF or Px, we adjusted its transcription rate accordingly. We also changed the noise of the target protein by altering its translation rate or degradation rate constant. For all these scenarios, the particle number of dimers and monomers are 300 and 100, respectively, from the aspect of the deterministic model. For the other two noise buffering method, there is no dimer but we adjusted the feedback or feedforward strength in the same ways as mentioned before. Figure 6 represents the COV of the target protein. Remarkably, the COV of the target protein in case of dimerization reaction (blue) is always the lowest.

Figure 6A,B showed how the noise-buffering methods dealing with various noise intensity of TF and Px, respectively. In the case without a noise-buffering method, the COV of the target protein (yellow) increased along with the incremental noise of TF or Px. The feedback control (green) and incoherent FFL (purple) attenuated noise for the various intensity of TF but failed to attenuate the noise from Px. Intriguingly, the incremental noise of Px showed only a slight effect in the case of the dimerization reaction (blue). This result further confirmed that the dimerization efficiently attenuated noise from the protein-ligand interaction. In Fig. 6C, the change of the translation rate constant of the target protein hugely affected the COV. In Fig. 6D, the incremental degradation rate constant of the target protein had a little influence on the COV in the case without a noise-buffering method but it severely undermined the noise attenuation of all three methods. We then try to increase the rate constants of dimerization reactions in both directions to enhance the noise attenuation, but COV was only reduced a little (Text S1).

## Discussion

We discovered a natural noise-buffering motif, the dimerization reaction (Fig. 1B). We demonstrated that dimerization reaction by itself notably attenuated the noise of the protein monomer (Fig. 2A). In comparison to the other two noise-buffering motifs, negative feedback control and the incoherent FFL, the motif of dimerization led to more noise reduction (Fig. 2B). The results also held true while maintaining the level of the protein monomer (Fig. 3). For a reaction network with direct interaction between the protein monomers and other ligands (Fig. 4), dimerization reduced the COV of the protein monomer though both the negative feedback control and the incoherent FFL failed to buffer noise (Fig. 5). Finally, we illustrated that the dimerization reaction attenuated noise from various sources and always showed the lowest COV of the target protein (Fig. 6).

In the present study, the dimerization reaction attenuated effectively the noise of the protein monomer. In nature, many systems have protein dimer but use protein monomers as a determinant in gene regulation. For example, both the systems of the glucocorticoid receptor (GRα) and G protein-coupled receptors (GPCRs) behave in this way. Unliganded GRα exists in the cytoplasm but moves into the nucleus when it binds to a ligand. The GRα monomer alone is capable of regulation. The GRα homodimer is not necessary for DNA binding to activate the gene^25^. GPCRs constitute the largest class of membrane receptors, which regulate immune responses^26^. Among the GPCRs, the CCR3 monomer is the minimal functional unit in signal transduction though class C are known to form homodimers^27^. Additionally, in class A, the coexistence of monomers and homodimers has also been documented^28,29^. For these systems, it is possible that the formation of the dimer is to reduce the random fluctuations of the monomer.

Figure 5 demonstrates that dimerization quickly responded to the rapid changes caused by Px and buffered the noise of the protein monomer. Neither feedback control nor the incoherent FFL could buffer noise. The incoherent FFL failed because sRNA cannot compensate random fluctuations from Px and from the interaction between proteins and ligands. Although the major advantage of feedback control is its generality, it cannot attenuate noise from a system with a short response time. Feedback control involves transcription and translation processes so it has a longer response time. While decreasing the degradation rate constant of Px to elongate its response time, the feedback control became capable of buffering noise (Fig. S4). Although the dimerization reaction properly attenuated the noise of the protein monomer, it had one major disadvantage. It reduced noise at the cost of consuming protein monomers. Nevertheless, we found that the formation of only one protein dimer could reduce the COV of protein monomers by 7.5%.

There are several reasons makes the dimerization capable of attenuating noise. We first borrow wisdom from Van Kampen. He points out that the bimolecular sink reaction causes less noise than the degradation of the monomer^31^ (Text S2). We can apply his explanation to our system, too. Both bimolecular sink reaction and the dimerization reaction have the following feature. When there is more monomer than the average, the reaction rate increased. On the other hand, the reaction rate decreased when the number of the monomer is below the average. This feature makes the level of monomer close to the average. We can also borrow wisdom from incoherent FFL^13^. When the fluctuation of sRNA and target RNA are highly correlated, the repression of sRNA on the translation of target RNA attenuates noise of the target protein. This concept is basically the same as that used in the active noise cancelling headphone. Because the trajectory of the repressor is similar to that of the target, it cancelled the noise of the target. In dimerization reactions, we may consider one monomer as a repressor and the other monomer as the target protein. These two monomers share the same trajectory. Thereby, dimerization reactions attenuate noise. Better than the incoherent FFL, which reduces only the extrinsic fluctuations, dimerization is able to attenuate the noise from the translation.

### Models

Figure 1 is the reaction networks with the nomenclature of the variables in Table 1. We examined four cases: a reaction network without any noise-buffering mechanism (Fig. 1A), the system with a dimerization reaction (Fig. 1B), the system with negative feedback control (Fig. 1C), and the system with the incoherent FFL in a prokaryote (Fig. 1D). When we further accounted for the interaction between proteins and ligands, the reaction networks became those presented in Figs 4, S1, S2 and S3. The nomenclature of the variables is in Table 1. The nominal values of parameters in Table S1 were adopted from the literature^13,32,33^. The nomenclature of additional variables is provided in Table S2. The reactions are presented in Tables S3–S6.

**Table 1:** Nomenclature.

Annotation	Description
*TF*	Transcription factor
*DNA*	Target gene
*DNA*_*X*_	Gene x
*DNAs*	Gene s
*RNA*	Target RNA
*RNAx*	The RNAx from DNAx
*sRNA*	The sRNA from DNAs
*P*	Target protein
*Px*	The peptide encoded in gene x
*P2*	Protein Dimer
*X*	Complex X from P bound with Px

While dealing with the reaction network shown in Fig. 1, we set the amount of Px to zero. We applied stochastic simulation algorithm (SSA)^34^ to each reaction network. The cellular volume was assumed to be 10^−15^ L. The initial conditions were zero for all intracellular variables, except for DNA, which was one per cell. We applied distribution to the time span between cell division by generating Gaussian random numbers. The standard deviation was 10% of the mean according to experimental observation^35^. The partition of intracellular species followed a binomial distribution^36^. We obtained the stationary distribution when the first and second moments of intracellular states became time independent. Specifically, we sampled the data at ninety thousand seconds. The distribution was composed of ten thousand trajectories, but the mean and variance were from one thousand trajectories at least. Based on the same reactions, we also formulated a deterministic model according to the law of mass action. The ordinary differential equations (ODEs) are provided in Tables S7–S10. We conducted steady-state analysis using Matlab functions fsolve or solve of the symbolic toolbox.

## Supplementary information

Supplementary Material
