# Author Correction: The Reaction of Dimerization by Itself Reduces the Noise Intensity of the Protein Monomer

**DOI:** 10.1038/s41598-020-58230-0

**Published:** 2020-01-24

**Authors:** Feng-You Liu, Shih-Chiang Lo, Che-Chi Shu

**Affiliations:** Department of Chemical Engineering and Biotechnology, National Taipei University of Technology, Taipei City, Taiwan R.O.C.

Correction to: *Scientific Reports* 10.1038/s41598-019-39611-6, published online 04 March 2019

In the Supplementary Material file originally published with this Article, text was omitted from the ‘Text S2’ section. This error has been corrected in the Supplementary Information that now accompanies the Article.

