# Supplementary material for: The Reaction of Dimerization by Itself Reduces the Noise Intensity of the Protein Monomer
Source: Sci Rep. 2019 Mar 4;9:3405. doi: 10.1038/s41598-019-39611-6 (PMC6399348; doi:10.1038/s41598-019-39611-6)
Supplement: Supplementary file 1 — Supplementary Material [file 41598_2019_39611_MOESM1_ESM.docx]

**The Reaction of Dimerization by Itself Reduces**

**the Noise Intensity of the Protein Monomer**

Feng-You Liu, Shih-Chiang Lo, Che-Chi Shu*

**Supplementary Materials**

(The followings are based on the order mentioned in the main text.)

| Table S1: The values of parameters | | | | |
| --- | --- | --- | --- | --- |
| Parameter | Description | Value | Units | Ref. |
| $k_{Rt}$ | Transcription rate constant of DNAt | 3.1x10^-3^ | S^-1^ | [1] |
| $k_{T}$ | Translation rate constant of RNAt | 7.8x10^-1^ | S^-1^ | [4] |
| $k_{drt}$ | Degradation rate constant of RNAt | 6x10^-3^ | S^-1^ | ^1^ |
| $k_{dt}$ | Degradation rate constant of TF | 2x10^-3^ | S^-1^ | ^1^ |
| $k_{R}$ | Transcription rate constant of DNA | 7.82x10^-3^ | S^-1^ | ^2^ |
| $k_{Ri}$ | Transcription rate constant of DNAs | 7.82x10^-3^ | S^-1^ | ^2^ |
| $k_{Rx}$ | Transcription rate constant of DNAx | 1.50x10^-4^ | S^-1^ | - |
| $k_{P}$ | Translation rate constant of RNA | 5x10^-3^ | S^-1^ | ^2^ |
| $k_{Px}$ | Translation rate constant of RNAx | 2.51x10^-1^ | S^-1^ | [4] |
| $k_{PP}$ | Binding rate constant of P2 | 1.37x10^5^ | M^-1^S^-1^ | ^3^ |
| $k_{X}$ | Binding rate constant of X | 10^6^ | M^-1^S^-1^ | [3] |
| $k_{RX}$ | Unbinding rate constant of X | 1.06x10^-1^ | S^-1^ | - |
| $k_{dr}$ | Degradation rate constant of RNA | 10^-4^ | S^-1^ | ^2^ |
| $k_{dri}$ | Degradation rate constant of sRNA | 10^-4^ | S^-1^ | ^1^ |
| $k_{drx}$ | Degradation rate constant of RNAx | 10^-4^ | S^-1^ | ^1^ |
| $k_{dp}$ | Degradation rate constant of P | 10^-6^ | S^-1^ | ^2^ |
| $k_{dpx}$ | Degradation rate constant of Px | 10^-6^ | S^-1^ | [1] |
| $k_{dpp}$ | Degradation rate constant of P2 | 10^-6^ | S^-1^ | ^2^ |
| $k_{dx}$ | Degradation rate constant of X | 10^-4^ | S^-1^ | ^2^ |
| $c$ | Hill coefficient | 2 | - | [2] |
| $w$ | Hill coefficient | 2 | - | [2] |
| $hr$ | Dissociation constant | 200 | number | [2] |
| $k_{RPP}$ | Unbinding rate constant of P2 | 5.40x10^-3^ | S^-1^ | * |
| $N$ | Constant specifies the quantity of sRNA that determines the translation rate of 0.5 | 5.65 | number | * |
| $K$ | Dissociation constant | 13.33 | number | * |

*We adjusted the values of these three parameters according to the statement in main text or figures.


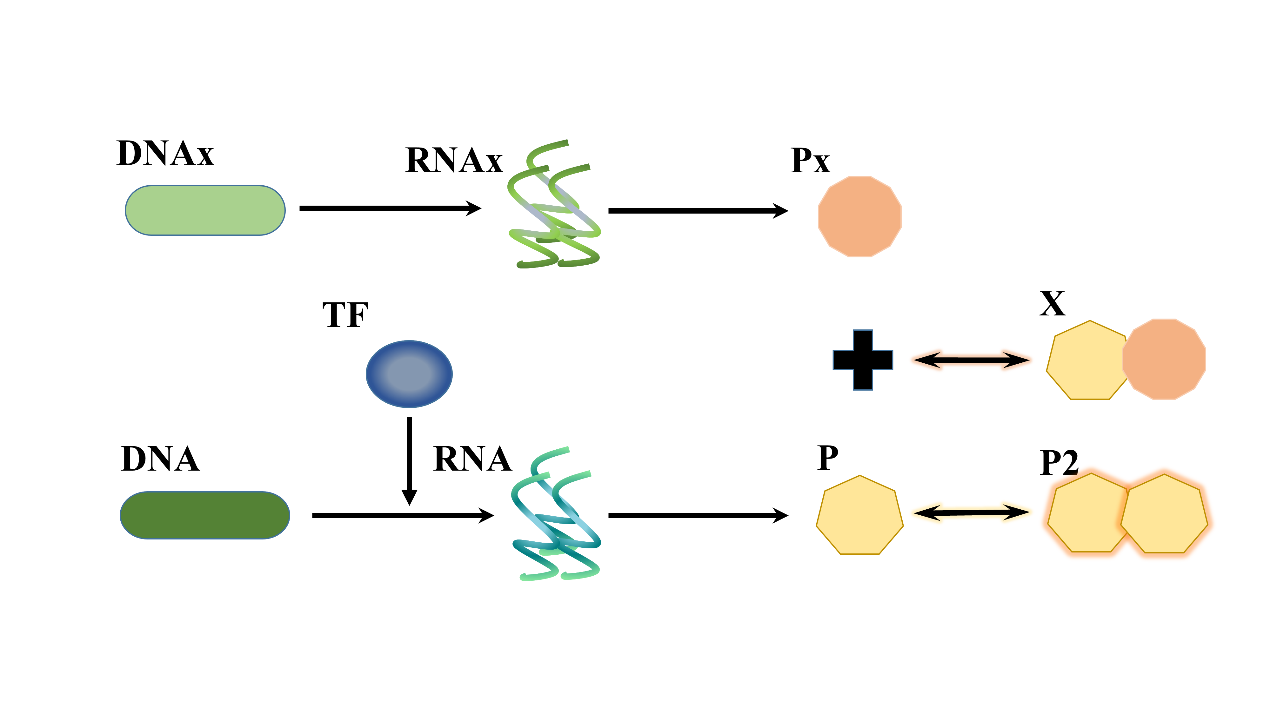


**Figure S1 The reaction network of the case with the reaction of dimerization and the direct interaction between the protein and ligand**

Based on the system in figure 1B, we further accounted for the direct interaction between the protein monomer and the ligand, Px.


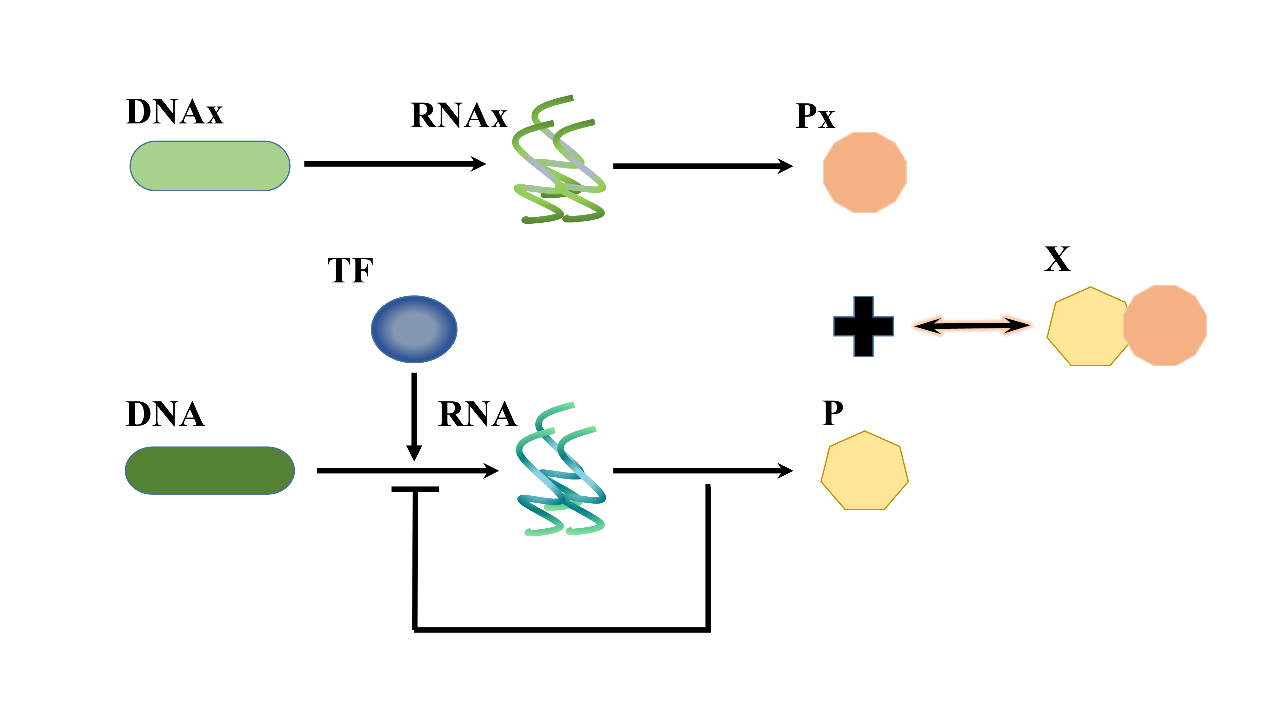
**Figure S2 The reaction network of the case with the negative feedback control and the direct interaction between the protein and ligand**

Based on the system in figure 1C, we further accounted for the direct interaction between the protein monomer and the ligand, Px.


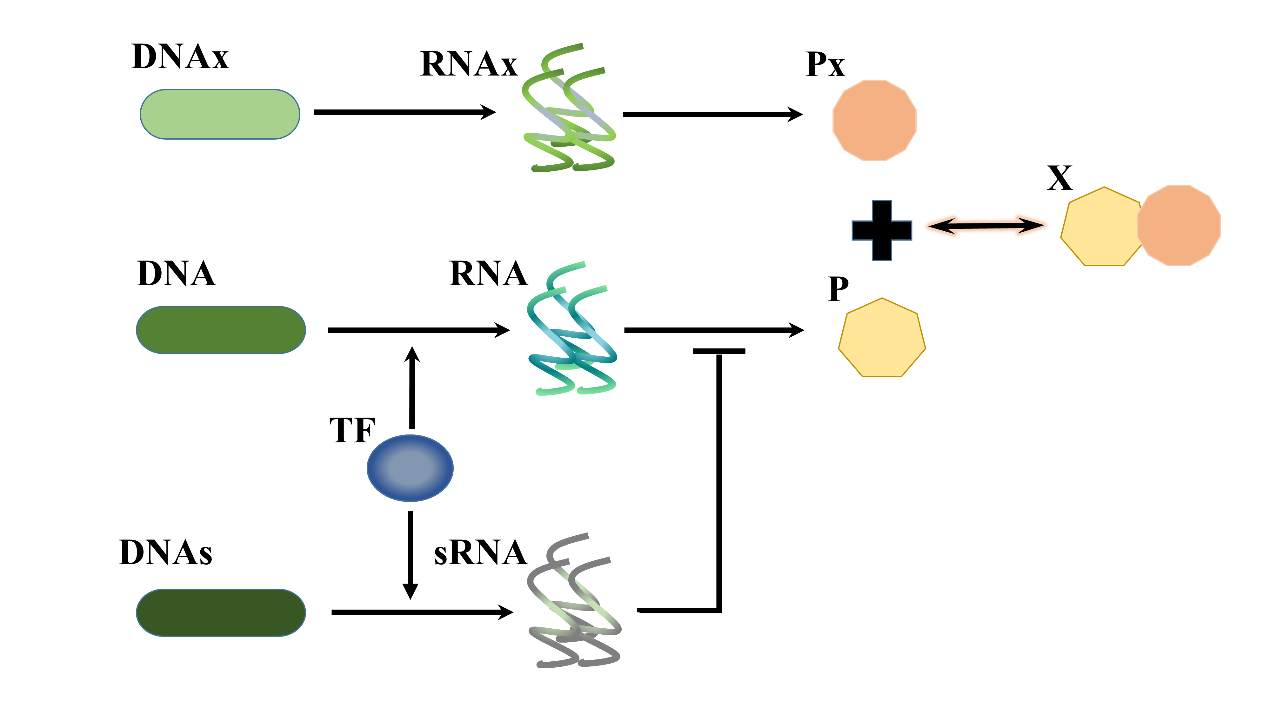
**Figure S3 The reaction network of the case with the incoherent FFL and the direct interaction between the protein and ligand**

Based on the system in figure 1D, we further accounted for the direct interaction between the protein monomer and the ligand, Px.

**Text S1**

In Fig 6D, the incremental degradation rate severely undermined the noise attenuation. To overcome it, we try to increase the rate constants of dimerization reactions in both directions. For protein degradation rate of 0.01, we decrease the COV from 0.360 to 0.336 as the rate constants were adjusted to 4 folds of nominal values. Intriguingly, the COV increased as we further increased the rate constants of dimerization reactions. When the rate constants were 8 and 10 folds of nominal values, the COV became 0.343 and 0.350, respectively.

**
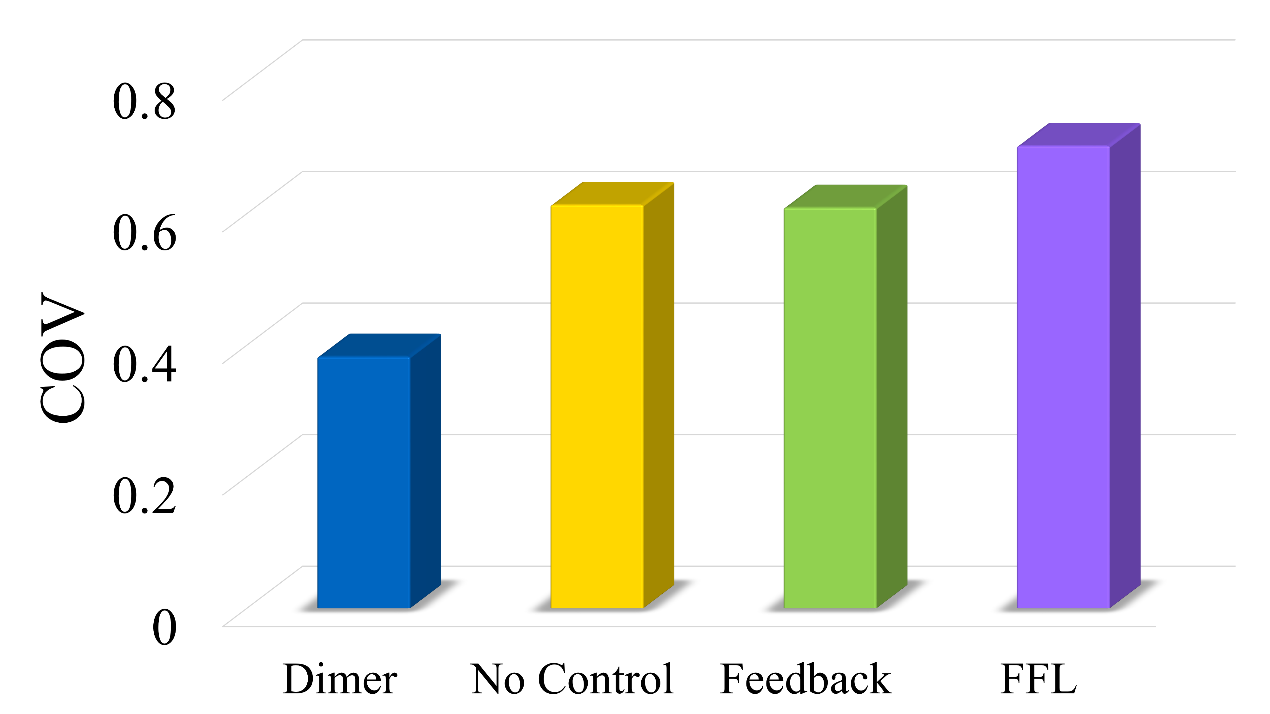
**

**Figure S4 The COV of the protein monomer in the system with the direct interaction of the protein and ligand**

The blue is for the case with the reaction of dimerization, yellow for the case without a noise buffering motif, green the case with a feedback control, and purple the case with incoherent FFL. While decreasing the degradation rate constant of Px to 1.00E-06 S^-1^ along with the translational rate constant of Px to 0.251 S^-1^ to keep the same level of Px, the feedback control became capable of reducing the noise of protein monomer.

**Text S2**

The equation (**1**.1) in Chapter X of Van Kampen’s book, Stochastic Processes in Physics and Chemistry^5^, describes equation set (S1). It includes a source reaction and a bimolecular sink reaction. He assumed A is continually supplied and B is drained.

| $A\underset{\to}{}X, 2X\underset{\to}{}B$ | (S1) |
| --- | --- |

He then compared equation set (S1) to equation set (S2). The later includes a generation reaction and a degradation reaction of monomer.

| $A\underset{\to}{}X, X\underset{\to}{}B$ | (S2) |
| --- | --- |

He found that the variance of X in equation set (S1) in only 3/4 of that in equation set (S2). Clearly, a bimolecular sink reaction is less noisy than a degradation reaction of monomer. It is quite an interesting finding. Nevertheless, this finding does NOT lead to a conclusion that dimerization reaction attenuates noise. Please allow me to explain it in the followings. We start from two general equation sets.

| $A\underset{\to}{}X, X\underset{\to}{1st\_Pathway}B$ | (S3) |
| --- | --- |
| $A\underset{\to}{}X, \boldsymbol{m}X\underset{\to}{2nd\_Pathway}B$ | (S4) |

The parameter m is case-dependent and will be specified later. Both equation sets (S3) and (S4) describe the source and sink reactions of X. We then discuss two cases.

In case I, we assigned a degradation reaction to $1st\_Pathway$ of equation set (S3). As for $2nd\_Pathway$ of equation set (S4), we assigned cell division to it so each time it causes the cell to lost nearly half of X according to a binomial random number. Namely, **m** in this case is a binomial random number. The noise of X in equation set (S3) is usually different from that of equation set (S4). Can we then claim that the pathway leads to less noise of X is a noise buffering motif ? No, of course not!! In fact, if we combined the $1st\_Pathway$and $2nd\_Pathway$ to have equation set (S5), equation set (S5) usually shows much higher noise than equation sets (S3) and (S4). The additional pathway causes more noise so it is NOT a noise-buffering motif.

| $A\underset{\to}{}X, X\underset{\to}{1st\_Pathway}B, \boldsymbol{m}X\underset{\to}{2nd\_Pathway}B$ | (S5) |
| --- | --- |

Before I moved to case II, I want to clearly state the conclusion we have in case I. There is not enough information to identify a noise-buffering motif from comparing the equation sets (S3) and (S4). This comparison merely tells us which pathway is less noisy.

In case II, we replaced **m** by **2** and assigned the bi-molecular sink reaction to $2nd\_Pathway$ so we have equation set (S4) in this case exactly the same as equation set (S1). If we assigned a degradation reaction to $1st\_Pathway$ of equation set (S3), the equation set (S3) in this case is exactly the same as equation set (S2). From the aforementioned conclusion in case I, we know that the comparison of equation set (S4) to equation set (S3) only give an information that the $2nd\_Pathway$ is less noisy. It does NOT imply the $2nd\_Pathway$ is a noise-buffering motif. Thereby, the finding in equation sets (S1) and (S2) does NOT lead to a conclusion that dimerization reaction attenuates noise.

| \| Table S2: The additional nomenclature \| \| \| --- \| --- \| \| Annotation \| **Description** \| \| *$DNAt$* \| The DNA encoding the TF \| \| *$RNAt$* \| The RNA from DNAt \| \| *$k_{Rtf}$* \| $\frac{k_{R}\times TF^{c}}{(hr^{c}+TF^{c})}$ \| \| *$k_{Ritf}$* \| $\frac{k_{Ri}\times TF^{c}}{(hr^{c}+TF^{c})}$ \| \| *$k_{Rtfk}$* \| $\frac{k_{R}\times TF^{c}\times K}{(hr^{c}+TF^{c})\times(K+P)}$ \| \| *$k_{Psr}$* \| $\frac{1}{1+(\frac{sRNA}{N})^{w}}$ \|   Table S3: The reactions of the system shown in Fig 4 |
| --- | --- | --- | --- | --- | --- | --- | --- | --- | --- | --- | --- | --- | --- | --- | --- | --- |
|  |
|  |
|  |
|  |
|  |
|  |
|  |
|  |
|  |
|  |
|  |
|  |
|  |
|  |

| Table S4: The reactions of the system with the reaction of dimerization and the direct interaction between the protein and ligand (Fig S1) |
| --- |
|  |
|  |
|  |
|  |
|  |
|  |
|  |
|  |
|  |
|  |
|  |
|  |
|  |
|  |
|  |
|  |

| Table S5: The reactions of the system with the negative feedback control and the direct interaction between the protein and ligand (Fig S2) |
| --- |
|  |
|  |
|  |
|  |
|  |
|  |
|  |
|  |
|  |
|  |
|  |
|  |
|  |
|  |

| Table S6: The reactions of the system with the incoherent FFL and the direct interaction between the protein and ligand (Fig S3) |
| --- |
|  |
|  |
|  |
|  |
|  |
|  |
|  |
|  |
|  |
|  |
|  |
|  |
|  |
|  |
|  |
|  |

| Table S7: The deterministic equations for the system without noise-buffering motif (shown in Fig 4). |
| --- |
| $\frac{d[RNAt]}{dt}=k_{Rt}[DNAt]-(\mu+k_{drt})[RNAt]$ |
| $\frac{d[TF]}{dt}=k_{T}[RNAt]-(\mu+k_{dt})[TF]$ |
| $\frac{d[RNA]}{dt}=\frac{k_{R}\times TF^{c}}{(hr^{c}+TF^{c})}[DNA]-(\mu+k_{dr})[RNA]$ |
| $\frac{d[RNAx]}{dt}=k_{Rx}[DNAx]-(\mu+k_{drx})[RNAx]$ |
| $\frac{d[P]}{dt}=k_{P}[RNA]-k_{X}[P][Px]+k_{RX}[X]-(u+k_{dp})[P]$ |
| $\frac{d[Px]}{dt}=k_{Px}[RNAx]-k_{X}[P][Px]+k_{RX}[X]-(u+k_{dpx})[Px]$ |
| $\frac{d[X]}{dt}=k_{X}[P][Px]-k_{RX}[X]-(u+k_{dx})[X]$ |

| Table S8: The deterministic equations for the system with the reaction of dimerization (shown in Fig S1). |
| --- |
| $\frac{d[RNAt]}{dt}=k_{Rt}[DNAt]-(\mu+k_{drt})[RNAt]$ |
| $\frac{d[TF]}{dt}=k_{T}[RNAt]-(\mu+k_{dt})[TF]$ |
| $\frac{d[RNA]}{dt}=\frac{k_{R}\times TF^{c}}{(hr^{c}+TF^{c})}[DNA]-(\mu+k_{dr})[RNA]$ |
| $\frac{d[RNAx]}{dt}=k_{Rx}[DNAx]-(\mu+k_{drx})[RNAx]$ |
| $\frac{d[P]}{dt}=k_{P}[RNA]-k_{X}[P][Px]+k_{RX}[X]-k_{PP}[P][P-1]+k_{RPP}[P2]-(u+k_{dp})[P]$ |
| $\frac{d[Px]}{dt}=k_{Px}[RNAx]-k_{X}[P][Px]+k_{RX}[X]-(u+k_{dpx})[Px]$ |
| $\frac{d[X]}{dt}=k_{X}[P][Px]-k_{RX}[X]-(u+k_{dx})[X]$ |
| $\frac{d[P2]}{dt}=k_{PP}[P][P-1]-k_{RPP}[P2]-(u+k_{dpp})[P2]$ |

| Table S9: The deterministic equations for the system with feedback control (shown in Fig S2). |
| --- |
| $\frac{d[RNAt]}{dt}=k_{Rt}[DNAt]-(\mu+k_{drt})[RNAt]$ |
| $\frac{d[TF]}{dt}=k_{T}[RNAt]-(\mu+k_{dt})[TF]$ |
| $\frac{d[RNA]}{dt}=\frac{k_{R}\times TF^{c}\times K}{(hr^{c}+TF^{c})\times(K+P)}[DNA]-(\mu+k_{dr})[RNA]$ |
| $\frac{d[RNAx]}{dt}=k_{Rx}[DNAx]-(\mu+k_{drx})[RNAx]$ |
| $\frac{d[P]}{dt}=k_{P}[RNA]-k_{X}[P][Px]+k_{RX}[X]-(u+k_{dp})[P]$ |
| $\frac{d[Px]}{dt}=k_{Px}[RNAx]-k_{X}[P][Px]+k_{RX}[X]-(u+k_{dpx})[Px]$ |
| $\frac{d[X]}{dt}=k_{X}[P][Px]-k_{RX}[X]-(u+k_{dx})[X]$ |

| Table S10: The deterministic equations for the incoherent FFL (shown in Fig S3). |
| --- |
| $\frac{d[RNAt]}{dt}=k_{Rt}[DNAt]-(\mu+k_{drt})[RNAt]$ |
| $\frac{d[TF]}{dt}=k_{T}[RNAt]-(\mu+k_{dt})[TF]$ |
| $\frac{d[RNA]}{dt}=\frac{k_{R}\times TF^{c}}{(hr^{c}+TF^{c})}[DNA]-(\mu+k_{dr})[RNA]$ |
| $\frac{d[RNAx]}{dt}=k_{Rx}[DNAx]-(\mu+k_{drx})[RNAx]$ |
| $\frac{d[sRNA]}{dt}=\frac{k_{Ri}\times TF^{c}}{(hr^{c}+TF^{c})}[DNAs]-(\mu+k_{dri})[sRNA]$ |
| $\frac{d[P]}{dt}=\frac{k_{P}}{1+(\frac{sRNA}{N})^{w}}[RNA]-k_{X}[P][Px]+k_{RX}[X]-(u+k_{dp})[P]$ |
| $\frac{d[Px]}{dt}=k_{Px}[RNAx]-k_{X}[P][Px]+k_{RX}[X]-(u+k_{dpx})[Px]$ |
| $\frac{d[X]}{dt}=k_{X}[P][Px]-k_{RX}[X]-(u+k_{dx})[X]$ |

**Reference**

# Jhang, W.-S., Lo, S.-C., Yeh, C.-C. & Shu, C.-C. Inhibitors Alter the Stochasticity of Regulatory Proteins to Force Cells to Switch to the Other State in the Bistable System. *Scientiftic Reports* 7, 4413 (2017).

# Osell, M., Bosia, C., Corá, D. & Caselle, M. The Role of Incoherent MicroRNA-Mediated Loops in Noise Buffering. *PloS one* 7, e1001101(2011).

# Markgren, P. O., Lindgren, M. T.,Gertow, K., Karlsson, R. Hämäläinen, M. & Danielson, U. H. Determination of Interaction Kinetic Constants for HIV-1 Protease Inhibitors Using Optical Biosensor Technology. *Analytical Biochemistry 291*, 207-218(2001).

# Shu, C.-C., Yeh, C.-C., Jhang, W.-S. & Lo, S.-C. Driving Cells to the Desired State in a Bimodal Distribution through Manipulation of Internal Noise with Biologically Practicable Approaches. *PloS one* 11, e0167563 (2016).

# Van Kampen, N. G. *Stochastic processes in physics and chemistry*. Vol. 1 (Elsevier, 1992).
